# Parkinson’s Disease: From Pathogenesis to Pharmacogenomics

**DOI:** 10.3390/ijms18030551

**Published:** 2017-03-04

**Authors:** Ramón Cacabelos

**Affiliations:** EuroEspes Biomedical Research Center, Institute of Medical Science and Genomic Medicine, 15165-Bergondo, Corunna, Spain; rcacabelos@euroespes.com

**Keywords:** adrenaline, antiparkinsonian drugs, Atremorine, dopamine, genomics, growth hormone, noradrenaline, Parkinson’s disease, pharmacogenetics, prolactin

## Abstract

Parkinson’s disease (PD) is the second most important age-related neurodegenerative disorder in developed societies, after Alzheimer’s disease, with a prevalence ranging from 41 per 100,000 in the fourth decade of life to over 1900 per 100,000 in people over 80 years of age. As a movement disorder, the PD phenotype is characterized by rigidity, resting tremor, and bradykinesia. Parkinson’s disease -related neurodegeneration is likely to occur several decades before the onset of the motor symptoms. Potential risk factors include environmental toxins, drugs, pesticides, brain microtrauma, focal cerebrovascular damage, and genomic defects. Parkinson’s disease neuropathology is characterized by a selective loss of dopaminergic neurons in the substantia nigra pars compacta, with widespread involvement of other central nervous system (CNS) structures and peripheral tissues. Pathogenic mechanisms associated with genomic, epigenetic and environmental factors lead to conformational changes and deposits of key proteins due to abnormalities in the ubiquitin–proteasome system together with dysregulation of mitochondrial function and oxidative stress. Conventional pharmacological treatments for PD are dopamine precursors (levodopa, l-DOPA, l-3,4 dihidroxifenilalanina), and other symptomatic treatments including dopamine agonists (amantadine, apomorphine, bromocriptine, cabergoline, lisuride, pergolide, pramipexole, ropinirole, rotigotine), monoamine oxidase (MAO) inhibitors (selegiline, rasagiline), and catechol-*O*-methyltransferase (COMT) inhibitors (entacapone, tolcapone). The chronic administration of antiparkinsonian drugs currently induces the “wearing-off phenomenon”, with additional psychomotor and autonomic complications. In order to minimize these clinical complications, novel compounds have been developed. Novel drugs and bioproducts for the treatment of PD should address dopaminergic neuroprotection to reduce premature neurodegeneration in addition to enhancing dopaminergic neurotransmission. Since biochemical changes and therapeutic outcomes are highly dependent upon the genomic profiles of PD patients, personalized treatments should rely on pharmacogenetic procedures to optimize therapeutics.

## 1. Introduction

Since 1915, over 85,000 papers have been published on Parkinson’s disease (PD) and related disorders in the international literature. The clinical entity described by James Parkinson (1755–1824) in 1817 as “paralysis agitans” in his “Assay on the Shaking Palsy” is at present the second most important neurodegenerative disorder in the elderly population, after Alzheimer’s disease.

With a prevalence ranging from 35.8 per 100,000 to 12,500 per 100,000 and annual incidence estimates ranging from 1.5 per 100,000 to 346 per 100,000 in different countries [1,2,3], PD is becoming a major age-related problem of health [4,5]. Meta-analysis of the worldwide data indicates a rising prevalence of PD with age (41 per 100,000 at 40–49 years; 107 at 50–59 years; 173 at 55–64 years; 428 at 60–69 years; 425 at 65–74 years; 1087 at 70–79 years; and 1903 per 100,000 at over age 80), also reflecting a characteristic distribution by geographic location (a prevalence of 1601 per 100,000 in patients from North America, Europe and Australia, and a prevalence of 646 per 100,000 in Asian patients [6]. Parkinson’s disease is more prevalent in males (1729 per 100,000, >65 years) than in females (1644 per 100,000), with a peak prevalence in the age group of ≥90 years (4633 cases per 100,000), and a mean prevalence of 1680 per 100,000 in people over 65 years of age [7]. Prevalence and incidence male/female (M/F) ratios increase by 0.05 and 0.14, respectively, per 10 years of age. Incidence is similar in men and women under 50 years (M/F ratio < 1.2), and over 1.6 times higher in men than women above 80 years [8]. Furthermore, PD coexists with dementia in over 25% of the cases and with depression in over 30% of the cases in some countries [7]. 

The diagnosis of PD basically relies on clinical data characterized by rigidity, resting tremor, bradykinesia and postural imbalance. Although the primary cause of neurodegenerative disorders and the pathogenic mechanisms underlying protein conformational changes and premature neurodegeneration are unknown, recent advances in genomic medicine (structural and functional genomics, epigenetics, transcriptomics, proteomics, metabolomics, pharmacogenomics) have greatly contributed to better understand the complex processes responsible for age-related neuronal death in neurodegenerative disorders [9,10,11,12,13,14].

From a therapeutic perspective, the introduction of levodopa (l-DOPA) in the 1960s represented a breakthrough in the treatment of PD, and it continues to be the most effective symptomatic therapy in Parkinsonian disorders [15]. However, the chronic administration of l-DOPA and other antiparkinsonian drugs currently causes severe side effects that deserve special attention by the medical community. In this regard, novel compounds devoid of psychomotor, biochemical, neuropsychiatric and autonomic complications are under experimental scrutiny in preclinical studies and clinical trials [16,17,18]. 

A substantial component of drug pharmacokinetics and pharmacodynamics in PD can be attributed to the pharmacogenetic profile of each patient [19] (Table 1). Pathogenic, mechanistic, metabolic, transporter and pleiotropic genes associated with the pharmacogenetic outcome are determinant in the optimization of PD therapeutics [17,18]. 

Important issues to take into consideration for the appropriate management of PD are the following: (i) identification of environmental factors responsible for PD-related neurotoxicity; (ii) characterization of the population at risk for developing PD with predictive biomarkers; (iii) implementation of preventive programs; (iv) optimization of therapeutics with conventional antiparkinsonian drugs; (v) development of novel compounds with specific neuroprotective effects on the dopaminergic system and devoid of severe side effects; and (vi) incorporation of pharmacogenetic procedures for a personalized treatment of PD patients [17,18,19,20].

## 2. Pathogenic Mechanisms

As in other prevalent age-related neurodegenerative disorders, it is plausible that the confluence of genomic vulnerability with diverse environmental factors may be responsible for the growing impact of PD in our society. Parkinson’s disease-related neurodegeneration is likely to occur several decades before the onset of the motor symptoms [20]. Associated with different potentially pathogenic risk factors (toxins, drugs, pesticides, brain microtrauma, focal cerebrovascular damage, genomic defects), PD neuropathology is characterized by a selective loss of dopaminergic neurons in the substantia nigra pars compacta and Lewy body deposition, with widespread involvement of other CNS structures and peripheral tissues [21,22,23]. Parkinson’s disease is a form of multisystemic α-synucleinopathy with Lewy bodies deposited in the midbrain. Descriptive phenomena to explain in part this neuropathological phenotype include the following: (i) genomic factors; (ii) epigenetic changes; (iii) toxic factors; (iv) oxidative stress anomalies; (v) neuroimmune/neuroinflammatory reactions; (vi) hypoxic-ischemic conditions; (vii) metabolic deficiencies; and (viii) ubiquitin–proteasome system dysfunction [19,23,24,25,26,27,28,29]; all these conditions leading to protein misfolding and aggregation and premature neuronal death. Recent evidence also suggests that PD might be a prion-like disease [30]. Telomere shortening as a result of the inability to fully replicate the ends of linear chromosomes is one of the hallmarks of aging which might also contribute to PD pathology [31]. 

Mutations in a series of primary genes are known to cause autosomal dominant and recessive forms of PD [28,29,30,31,32,33,34,35,36,37,38]. Mutations in some genes—e.g., α-Synuclein (*SNCA*), Parkin 2 (*PARK2*), PTEN-induced putative kinase 1 (*PINK1*), *PARK7*, Leucine-rich repeat kinase 2 (*LRRK2*), Bone narrow stromal cell antigen 1 (*BST1*), Microtubule-associated protein tau (*MAPT*)—might be causative in familial forms of PD whereas diverse genetic defects in other loci might represent susceptibility loci associated with sporadic PD without family history [28,34,35,36,37,38,39]. Mendelian variants with high penetrance (e.g., *SNCA*, *LRRK2*, *PINK1*, *PARK7* genes) explain less than 10% of familial PD [40]. For the past decade several genome-wide association studies (GWAS) have contributed to clarify the contribution of genetic factors to the pathogenesis of PD in the Caucasian population and in other ethnic groups [41,42,43,44,45,46,47,48,49,50,51]. In a recent meta-analysis of PD GWAS with over 7 million variants, 26 loci have shown significant association with PD. Replication studies confirmed 24 single nucleotide polymorphisms (SNPs), and conditional analyses within loci showed four loci —β-Glucocerebrosidase (*GBA*); Diacylglycerol kinase θ, 110kD (*GAK-DGKQ*); *SNCA*; Human leukocyte antigen (*HLA*)—with a secondary independent risk variant [52]. Significant associations at different loci—Discs large homolog of 2 (*DLG2*), Sipa1-like protein (*SIPA1L2*), Serine/Theonine kinase 39 (*STK39*), Vacuolar protein sorting 13 homolog C (*VPS13C*), Ric-like protein without CAAX motif 2 (*RIT2*), *BST1*, *PARK16*—have been found in Asians vs. Europeans, together with allelic heterogeneity at *LRRK2* and at six other loci including *MAPT* and *GBA-SYT11* [50]. Some other candidate genes have been recently reported to be associated with PD in different cohorts (i.e., *RAD51B* [39], *DYRK1A* [53], *CHCHD2* [54], *VPS35* [55], *RAB39B* [56], *TMEM230* [57]).

In addition to the mutations of primary genes responsible for PD-related synucleopathy, most defective loci identified so far are linked to pathogenic pathways leading to premature neurodegeneration in PD [51]. α-Synuclein accumulation, mitochondrial dysfunction, autophagic impairment, and oxidative and endoplasmic reticulum stress are common findings in the PD pathogenic cascade. The *SNCA* (α-synuclein) gene encodes a presynaptic protein that tends to misfold and is subsequently found to be a major component of Lewy bodies, a histological hallmark of the PD brain. Point mutations in the *SNCA* gene cause rare familial forms of PD. Duplication/triplication of the wild type *SNCA* gene also causes a form of PD, indicating that increased levels of the normal α-synuclein protein are sufficient to cause the disease. Several SNPs in the *SNCA* gene are associated with an increased risk of developing sporadic PD. A GWAS has identified a novel SNP rs356182 at *SNCA* that can modulate the risk of PD in Caucasian ancestry and in the Chinese population [58]. 

Glucocerebrosidase 1 (GBA1) mutations, resulting in misfolded glucocerebrosidase (GCase), affect the functioning of endoplasmic reticulum (ER), lysosomes, and mitochondria. Misfolded GCase trapped in the ER leads to an increase in both the ubiquitin–proteasome system (UPS) and the ER stress. The presence of ER stress triggers the unfolded protein response (UPR) and/or ER-associated degradation, with subsequent increase in apoptosis. The presence of misfolded GCase in the lysosomes together with a reduction in wild type GCase levels leads to a retardation of α-synuclein degradation via chaperone-mediated autophagy, which results in α-synuclein accumulation and aggregation. Impaired lysosomal functioning also causes a decrease in the clearance of autophagosomes, and accumulation of this cellular detritus. *GBA1* mutations perturb normal mitochondria functioning by increasing the generation of free radical species (ROS) and decreasing ATP production, oxygen consumption, and membrane potential [59]. Furthermore, heterozygous *GBA1* mutations alter the lysosomal enzyme that converts glucosyl-ceramides into ceramide, and increase the risk of developing PD [60]. Defects in ceramide metabolism have been recognized in PD. Altered sphingolipid composition in Lrrk2^−/−^ mouse brains has been observed in lipidomic studies. Ceramide levels are increased in Lrrk2^−/−^ mice with effects on GBA1 [60].

Oxidative stress plays a central role in the progress of PD (i) affecting nucleic acid stability by oxidizing RNA, increasing mitochondrial DNA (mtDNA) mutation, and launching translesion synthesis (TLS); (ii) disturbing protein homeostasis by accelerating α-synuclein aggregation, parkin aggregation, and proteasome dissociation; (iii) modulating dopamine release by activating ATP-sensitive potassium channels (K_ATP_) and inactivating neuronal nicotinic acetylcholine receptors (nAChRs); and (iv) influencing cellular self-defenses by promoting the cytoprotective effects of oncogene DJ1(DJ-1) and phosphatase and tensin homolog PINK1 while inducing Akt dysregulation [53,61,62]. Elevated levels of oxidative or nitrative stresses have been implicated in α-synuclein-related toxicity. Phosphorylation of α-synuclein on serine 129 (S129) modulates autophagic clearance of inclusions and is prominently found in Lewy bodies [63]. Mutations in the *LRRK2* gene associated with PD also cause autophagy dysregulation [64]. Some studies delineating the pathways involved in parkin/PINK1-mediated mitophagy also contributed to the renaissance of the “mitochondrial theory of PD” [65,66]. Dysfunction of the mitochondria caused by bioenergetic defects, mutations in mitochondrial DNA, nuclear DNA gene mutations linked to mitochondria, and changes in dynamics of the mitochondria such as fusion or fission, changes in size and morphology, alterations in trafficking or transport, altered movement of mitochondria, impairment of transcription, and the presence of mutated proteins associated with mitochondria are implicated in PD [66]. 

The “proteasome theory of PD” is based on the fact that several genes with polymorphic variants associated with different forms of PD encode proteins which are integrated in the machinery of the proteasome system. Parkin is a unique, multifunctional ubiquitin ligase whose various roles in the cell, particularly in neurons, are widely thought to be protective. Parkin dysfunction represents a predominant cause of familial parkinsonism and also a formal risk factor for the sporadic form of PD. Parkin exerts housekeeping protein quality control roles, and regulates mitochondrial homeostasis and stress-related signaling. Parkin functions as an E2-dependent ubiquitin ligase associated with the UPS, a major intracellular protein degradation machinery. Parkin is also a key mammalian regulator of mitochondrial autophagy (mitophagy). Neurodegeneration in PD patients harboring homozygous loss-of-function mutations in the *PARK2* gene may result from unbalanced levels of ROS, which are mostly produced in mitochondria and can irreparably damage macromolecules and trigger apoptosis [67]. Mutations in genes encoding proteins that interact with parkin in the UPS may also contribute to PD. Mutations in *PINK1* cause early onset familial PD. PINK1 accumulates on the outer membrane of damaged mitochondria followed by recruiting parkin to promote mitophagy [65]. Mutations in the *FBX07* (F-box protein 7) gene, encoding an adaptor protein in Skp–Cullin–F-box (SCF) SCF(FBXO7) ubiquitin E3 ligase complex, to recognize substrates and mediate substrate ubiquitination by SCF(FBXO7) E3 ligase, are also associated with PD. Parkinson’s disease-linked FBXO7 can recruit parkin into damaged mitochondria and facilitate its aggregation [68]. Point mutations within *FBXO7* map within specific functional domains, including near its F-box domain and its substrate recruiting domains, suggesting that deficiencies in SCF^Fbxo7/PARK15^ ubiquitin ligase activity are mechanistically linked to early-onset PD. FBXO7 negatively regulates glycogen synthase kinase 3 β (GSK3β) activity. FBXO7 ubiquitinates translocase of outer mitochondrial membrane 20 (Tomm20), and its levels correlate with Fbxo7 expression, indicating a stabilizing effect [69]. DJ-1, the product of the causative gene of a familial form of PD, plays a significant role in anti-oxidative defense to protect cells from oxidative stress. DJ-1 undergoes preferential oxidation at the cysteine residue at position 106 (Cys106) under oxidative stress. oxDJ-1 is transformed into dimer and polymer forms that interact with 20S proteasome [70]. 

Proteasome subunits (PSMB) and transporter associated with antigen processing (TAP) loci located in the HLA class II region play important roles in immune response and protein degradation in neurodegenerative diseases. Immune dysregulation, which was associated with the major histocompatibility complex (MHC) class I pathway, may be involved in PD pathogenesis. Proteasome subunits and TAP genes are responsible for immune activity and protein degradation in MHC class I pathway. The classical MHC class molecules, HLA-DRB, represent the antigens for immune effector cells associated with PD. The *PSMB* and *TAP* genes are adjacent to HLA-DR within the HLA class II region. *PSMB9* and *PSMB8* encode β1i (low molecular weight protein 2) and β2i (low molecular weight protein 7) subunits of immunoproteasome, and replace the proteasome subunits in the ubiquitin–proteasome system as “immune” subunits. *PSMB* and *TAP* genes may be involved in α-synuclein degradation with more genetic susceptibility to PD by regulation of immunoproteasome in dopaminergic neurons [71]. 

Defects in genes responsible for pesticide metabolism (PON1), transport across the blood–brain barrier (ABCB1), pesticides interfering with or depending on dopamine transporter activity (DAT/SLC6A3) and dopamine metabolism (ALDH2), impacting mitochondrial function via oxidative/nitrosative stress (NOS1) or proteasome inhibition (SKP1), and contributing to immune dysregulation (HLA-DR), may also represent additional risk factors for PD [22]. 

Parkinson’s disease-related pathogenic and susceptibility genes are under the influence of the epigenetic machinery (DNA methylation, chromatin remodeling, histone modifications, miRNA regulation) that regulates their expression in different tissues and may contribute to selective nigrostriatal dopaminergic neurodegeneration. Epigenetic changes in PD-related genes also contribute to PD pathogenesis and epigenetic drugs might constitute a potential therapeutic option in the future [26,34,35,72,73,74,75,76,77].

## 3. Conventional Treatments

Classical therapeutic interventions for the symptomatic treatment of psychomotor dysfunction in PD include pharmacotherapy, deep brain stimulation, and physiotherapy [78]. In addition to dopamine precursors (l-DOPA), other symptomatic treatments for PD include dopamine agonists (amantadine, apomorphine, bromocriptine, cabergoline, lisuride, pergolide, pramipexole, ropinirole, rotigotine), monoamine oxidase (MAO) inhibitors (selegiline, rasagiline), and catechol-O-methyltransferase (COMT) inhibitors (entacapone, tolcapone) [9,15,17,19] (Table 1). The initial complication of long-term l-DOPA therapy is the “wearing-off” phenomenon [79,80], together with motor fluctuations and dyskinesia, which develop during the use of both l-DOPA and dopamine agonists [15,81]. Diverse dopaminergic and nondopaminergic pharmacological approaches have been developed to manage such complications, including novel l-DOPA formulations, COMT inhibitors (opicapone), dopamine agonists, adenosine A2A antagonists (istradefylline, preladenant, tozadenant), glutamatergic *N*-methyl-d-aspartate (NMDA) antagonists, serotonergic agents (eltoprazine), and metabotropic glutamate receptor 5 (mGluR5) modulators (mavoglurant), with controversial results [82,83]. Polypharmacy with antidepressants, antipsychotics, urological drugs, analgesics, antihistaminics and cholinesterase inhibitors also contributes to severe complications associated with the anticholinergic burden in PD [84]. Furthermore, gastrointestinal complications (constipation, sialorrhea, dysphagia, difficulty in mastication, choking/aspiration) [85,86], cardiovascular problems [87,88], neuroendocrine changes and psychiatric disorders are frequent in PD patients chronically treated with conventional antiparkinsonian drugs [9,85]. An additional complication is the Pisa syndrome, defined as a reversible lateral bending of the trunk with a tendency to lean to one side [89]. It is common to administer atypical neuroleptics (e.g., quetiapine) when PD patients develop psychotic symptoms. This pharmacological intervention should be avoided due to the fact that most neuroleptics display an antidopaminergic effect which may neutralize or reduce the pharmacological action of antiparkinsonian drugs, contributing to additional psychomotor deficits.

## 4. Pharmacogenomics

Pharmacogenomics accounts for 60%–90% variability in the pharmacokinetics and pharmacodynamics of antiparkinsonian drugs. Genes involved in the pharmacogenetic network include pathogenic, mechanistic, metabolic, transporter and pleiotropic genes [9,10,11,12,13,14] (Table 1), and all these genes are also under the influence of epigenetic modifications (DNA methylation, histone/chromatin remodeling, mRNA regulation) [12,13,14]. In recent years novel evidence has demonstrated the impact of pharmacogenetics on antiparkinsonian drug efficacy and safety [9,17,90,91,92,93] (Table 1). In the particular case of l-DOPA, the *ANKK1*, *BDNF*, *LRRK2*, and *PARK2* genes are pathogenic genes potentially involved in its effects. The *CCK*, *CCKAR*, *CCKBR*, *DRD1*, *DRD2*, *DRD3*, *DRD4*, *DRD5*, *GRIN2A*, *GRIN2B*, *HCRT*, *HOMER1*, *LMO3*, and *OPRM1* genes are mechanistic genes whose products influence l-DOPA efficacy and safety. l-DOPA is a substrate of enzymes encoded by the *COMT*, *CYP1A2*, *CYP2B6*, *CYP2C19*, *CYP2D6*, *CYP3A4*, *CYP3A5*, *DBH*, *DDC*, *G6PD*, *MAOB*, *TH*, *UGT1A1*, and *UGT1A9* genes responsible for its metabolism. Solute carrier family 6 member 3 (SLC6A3) is the major transporter of l-DOPA and *ACE*, *ACHE* and *APOE* are pleiotropic players in l-DOPA efficacy and safety [9] (Table 1). *ADORA2A* SNPs and *HOMER1* variants may be associated with l-DOPA-induced dyskinesia and psychotic symptoms [94,95]. A haplotype integrating −141C Ins/Del, rs2283265, rs1076560, C957T, TaqIA and rs2734849 polymorphisms at the *DRD2*/*ANKK1* gene region might also be associated with l-DOPA-induced motor dysfunction [96]. SLC6A3 is a genetic modifier of the treatment response to l-DOPA in PD [97]. The multidrug resistance gene (*MDR1*) C1236T polymorphism may also influence PD pharmacotherapy [98] as well as SNPs in genes encoding the dopamine transporter (DAT; SLC6A3) and the vesicular monoamine transporter 2 (VMAT2; SLC18A2) [99]. Consequently, the personalized treatment of PD patients requires the implementation of pharmacogenetic procedures in order to optimize therapeutics (improving efficacy and safety) [9].

## 5. Novel Treatments

**Figure 1:**
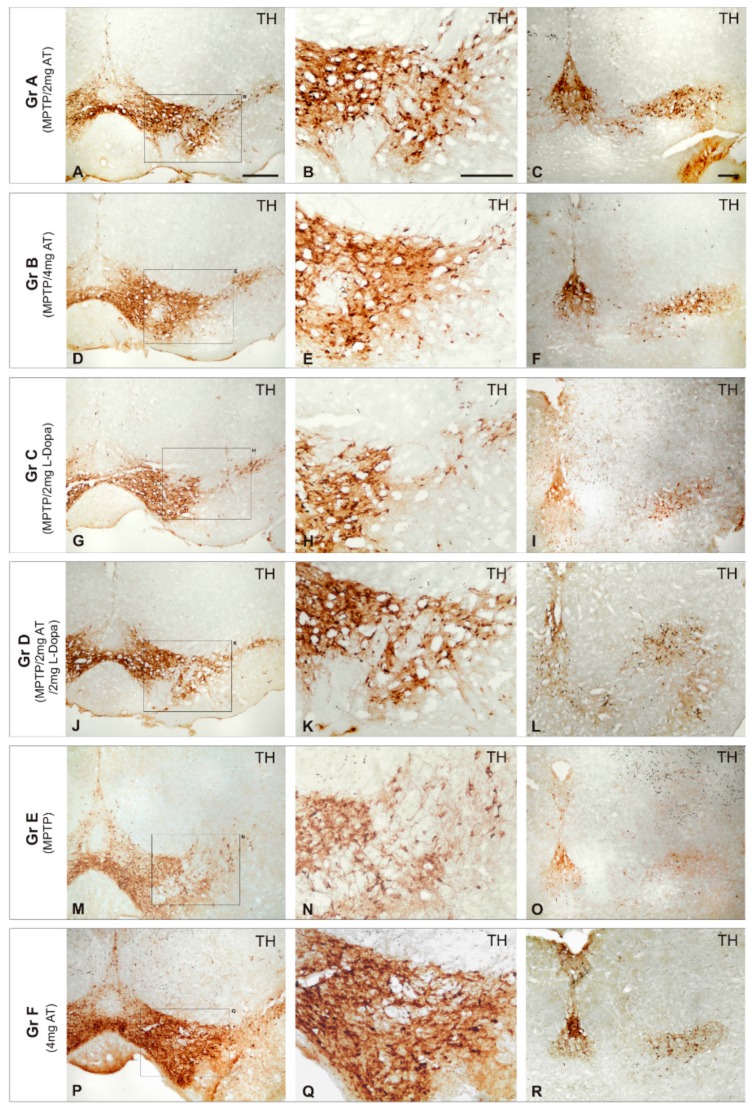
Effect of Atremorine (AT) treatment against dopaminergic degeneration. Comparative photomicrographs of tyrosine hydroxylase (TH) immunoreactivity were taken in the substantia nigra (SN) of mice with 1-methyl-4-phenyl-1,2,3,6-tetrahydropyridine (MPTP)-induced neurotoxicity, untreated (**E**) and treated with l-DOPA (**C**,**D**) or Atremorine (**A**,**B**). Note the remarkable effect of Atremorine (**A**,**B**) in reversing the neurotoxic effect of MPTP on dopaminergic neurons. (**A**–**F**) Atremorine; (**J**–**L**) Atremorine + L-DOPA; (**M**–**O**) Untreated; (**P**–**R**) Control + Atremorine. Scale bar: 100 µm. Adapted with permission from Carrera et al. [101].

The unsatisfactory effects of conventional antiparkinsonian drugs have prompted the search for novel alternatives. Some attempts have been made with novel compounds (alternative and complementary medicines) for PD in recent times [17,18,19,100,101]. The revival of some classic and novel natural products (i.e., *Vicia faba*, *Mucuna pruriens*, *Siegesbeckia pubescens*, *Sophora flavescens*, *Carthamus tinctorius*, *Curcuma longa*, *Huperzia selago*, *Diphasiastrum complanatum*, *Erythrina velutina*, *Gynostemma pentaphyllum*, Echinacoside, *Centella asiatica*, Sulforaphane, Oleuropein, flavonoids, Ginsenosides, cyanidin-3-*O*-glucoside, caffeine, Resveratrol, and other polyphenols) has also been proposed; and new applications have been submitted to the USA and European Patent Offices in this regard. Some of these natural products with potential efficacy in PD show selective dopaminergic neuroprotection to prevent neuronal death, with additional benefits such as antioxidant, anti-inflammatory, and neurotrophic effects. An example of this is E-PodoFavalin-15999 (Atremorine), a novel biopharmaceutical compound, obtained by means of non-denaturing biotechnological procedures from structural components of *Vicia faba* L., for the prevention and treatment of PD [17,18,19,101,102]. Preclinical studies (in vitro) revealed that Atremorine is a powerful neuroprotectant in (i) cell cultures of human neuroblastoma SH-SY5Y cells; (ii) hippocampal slices in conditions of oxygen and glucose deprivation; and (iii) striatal slices under conditions of neurotoxicity induced by 6-hydroxydopamine (6-OHDA). In vivo studies showed that Atremorine (i) protects against 1-methyl-4-phenyl-1,2,3,6-tetrahydropyridine (MPTP)-induced dopaminergic neurodegeneration; (ii) inhibits MPTP-induced microglia activation and neurotoxicity in the substantia nigra; and (iii) improves motor function in mice with MPTP-induced neurodegeneration [17,18,19,101,102] (Figure 1). 

**Figure 2:**
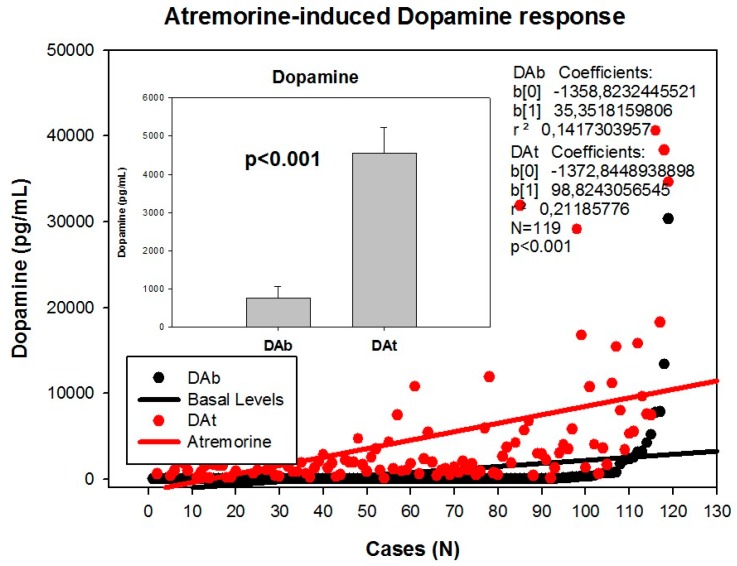
Atremorine-induced dopamine (DA) response in patients with Parkinsonian disorders. DAb: Basal dopamine levels; DAt: Plasma dopamine levels one hour after Atremorine administration (5 g, p.o.). Adapted with permission from Cacabelos et al. [18].

Clinical studies in untreated patients who receive Atremorine for the first time (never treated before with antiparkinsonian drugs) revealed that Atremorine enhances dopaminergic neurotransmission and increases plasma dopamine levels by 200–500-fold [17] (Figure 2). In patients chronically treated with l-DOPA or other antiparkinsonian drugs, Atremorine induces a dopamine response of similar magnitude to that observed in previously untreated patients. This pro-dopaminergic effect can be attributed to the rich content of natural l-DOPA (average concentration 20 mg/g) in the composition of Atremorine. However, the neuroprotective effect of this nutraceutical product on dopaminergic neurons, as demonstrated in in vitro studies and in animal models of PD [17,101,102], cannot be attributed to l-DOPA alone, but to other intrinsic constituents (selective neurotrophic factors) of the compound [17]. One hundred percent ofUntreated PD patients exhibit a dramatic hypodopaminemia, with plasma levels of dopamine (DA) below 20 pg/mL, and PD patients under long-term treatment with l-DOPA and/or conventional antiparkinsonian drugs experience a hyperdopaminemic status which might be responsible for (i) the clinical improvement of PD cardinal symptoms in the short term; (ii) the “wearing-off” phenomenon [79,80]; (iii) motor fluctuations and dyskinesia [15,81]; (iv) systemic complications (gastrointestinal disorders, cardiovascular problems, hormonal dysregulation) [85,86,87]; and (v) neuropsychiatric disorders (depression, anxiety, toxic psychosis) [9,103].

Atremorine is an option to minimize the “wearing-off” phenomenon, extending the therapeutic effect of conventional antiparkinsonian drugs, and reducing potential side effects, since the co-administration of Atremorine with other antiparkinsonian drugs allows a dose reduction of conventional drugs by 25%–50% with enhancement of clinical benefits and reduction of short- and long-term adverse drug reactions [19].

Atremorine is a powerful enhancer of plasma catecholamines (noradrenaline, adrenaline, dopamine), with no apparent effect on serotonin [18]. Catecholamines are processed by three main nuclei (A8-retrobulbal, A9-substantia nigra pars compacta, A10-ventral tegmental area) arranged in the mesencephalic region where the mesostriatal, mesolimbic and mesocortical pathways are organized [104,105]. Midbrain dopaminergic neurons in the ventral tegmental area and noradrenergic neurons in the locus coeruleus are major sources of dopamine and noradrenaline to the prefrontal cortex, where these amines regulate cognition, behavior, and psychomotor function [106,107]. Noradrenaline, adrenaline, dopamine and serotonin play a central role in CNS and gut pathophysiology. Dopamine and noradrenaline are involved in the chemical structure of neuromelanins in the substantia nigra and the locus coeruleus, respectively. Dopamine, 3,4-dihydroxyphenylacetic acid (DOPAC), 3,4-dihydroxyphenylethanol (DOPE), and 3,4-dihydroxyphenylalanine (DOPA) are mainly responsible for the structure of neuromelanin from substantia nigra, while noradrenaline, 3,4-dihydroxymandelic acid (DOMA), and 3,4-dihydroxyphenylethylene glycol (DOPEG) are responsible for the structure of neuromelanin from locus coeruleus [108]. Deficiencies in these monoamines are currently found in PD [109,110]. Monoamine transporters (MATs) that facilitate the reuptake of noradrenaline, dopamine, and serotonin are sodium-coupled transport proteins belonging to the neurotransmitter/Na^+^ symporter (NSS) family, which have also been implicated in PD [111,112]. Hypoactivity of the dopaminergic and noradrenergic systems in the brain stem are related to non-motor and motor symptoms in PD [113,114]. Dysregulation of these neurotransmitters is also involved in a variety of gastrointestinal symptoms in PD [115], and all of them appear to contribute to neurotransmitter and autonomic dysfunctions in PD [113], including mechanisms of l-DOPA-induced dyskinesia [115,116] and cardiovascular dysautonomia [117]. Therefore, appropriate doses of Atremorine alone or in combination with low doses of conventional antiparkinsonian drugs [118] may benefit PD patients in whom the biosynthetic apparatus of the catecholaminergic system is damaged, including tyrosine hydroxylase (TH), the tetrahydrobiopterin (BH4) cofactor of TH, and the activity of the BH4-synthesizing enzyme, GTP cyclohydrolase I (GCHI), as well as the activities of aromatic l-amino acid decarboxylase (AADC, DOPA decarboxylase), DBH, and phenylethanolamine *N*-methyltransferase (PNMT), which synthesize dopamine, noradrenaline, and adrenaline, respectively [119]. Atremorine may also neutralize the apoptosis of nigrostriatal dopamine neurons which, in post-mortem studies, show increased levels of pro-inflammatory cytokines—tumor necrosis factor-α (TNF-α), interleukin-6 (IL-6)—, increased levels of apoptosis-related factors—p 55, soluble Fas, B-cell lymphoma 2 (Bcl-2), caspases 1-2—, and decreased levels of neurotrophins (BDNF) [119].

The increase in noradrenaline induced by Atremorine may contribute to clinical improvement and neuroprotection, since noradrenergic neuronal loss in the locus coeruleus is exacerbated in PD. Lewy pathology in the locus coeruleus, the brain’s main source of noradrenaline, precedes that of the substantia nigra and may be one of the very first pathogenic events in PD. Oxidized noradrenaline exerts a neuroprotective effect and may even prevent the formation of toxic and higher molecular weight α-synuclein oligomers associated with PD. Noradrenergic neurons innervate the substantia nigra. The locus coeruleus orchestrates the other major catecholaminergic nuclei, such as the substantia nigra and raphe nuclei. In this regard, it has been suggested that neuronal loss in the locus coeruleus and the accompanying noradrenergic deficiency constitute an important pharmacological target for the treatment of PD [120]. Noradrenaline exerts critical effects in the modulation of different types of behavior (sleep-wakefulness cycle, depression, anxiety), psychomotor function, anti-inflammatory responses in glial cells, neurotrophic activity, and neuroprotection against oxidative stress-related free radical formation [121,122]. Preclinical studies showed that Atremorine displays powerful antioxidant, anti-inflammatory, and neuroprotective effects [102], some of which might be associated with its effect as a noradrenergic enhancer. Since premature noradrenaline deficiency resulting from selective degeneration of neurons of the locus coeruleus and sympathetic ganglia may be a praecox event in PD [123,124], the noradrenergic effects of Atremorine may also explain, in part, its clinical and biochemical benefits [17,18]. 

The midbrain dopaminergic system is regulated by the central adrenergic system [125]. The moderate increase in adrenaline levels observed after Atremorine administration may also contribute to enhancing dopaminergic neurotransmission in PD [18]. 

Neuroendocrine dysfunction and alterations in circadian rhythms are frequently seen in patients with PD, but most results are contradictory, with no clear definition between basal conditions and drug-induced modifications in hypothalamus-pituitary neuropeptides and hormones [126,127,128]. Furthermore, prolactin and growth hormone (GH) secretion are directly regulated by hypothalamic and supra-hypothalamic dopaminergic mechanisms [129,130,131]. Some studies reported higher levels of prolactin and GH in PD patients as compared with controls [128,132]. In patients with multiple system atrophy, in whom there is a reported loss of hypothalamic dopamine, basal prolactin levels are elevated, l-DOPA increases GH secretion, and the neuroendocrine response to l-DOPA is unclear [133], differing from endocrine responses in PD patients [134,135]. 6-Pyruvoyl-tetrahydropterin synthase (PTPS) deficiency is a BH4 deficiency with hyperphenylalaninemia, which is treated with l-DOPA/Carbidopa, 5-hydroxytryptophan (5-HTP) and BH4. In these patients, serum prolactin levels are elevated due to their hypodopaminergic condition, and the administration of l-DOPA reduces prolactin secretion [136]. In healthy subjects, acute l-DOPA and exercise release GH, but in PD patients this response is delayed [137]. In animal studies, l-DOPA causes a decrease in prolactin response, whereas cortisol levels tend to increase [138]. It has also been postulated that peripheral noradrenergic terminals may contribute to regulating prolactin secretion [139].

Atremorine induces a significant decrease in prolactin, GH, and cortisol levels [18]. The prolactin and GH response to Atremorine can be directly attributed to the effect of l-DOPA on dopamine and noradrenaline synthesis and release, with the consequent increase in central and peripheral dopamine and noradrenaline levels. In contrast, the effect on cortisol might be primarily influenced by a direct effect of dopamine, noradrenaline and adrenaline on the adrenal gland, and secondarily by pituitary and/or hypothalamic regulation of adrenocorticotropic hormone (ACTH), which in plasma did not show any significant changes. Neuroendocrine function in PD is still poorly understood, and the investigation of differences in PD-related basal neuroendocrine conditions vs antiparkinsonian drug-induced neuroendocrine changes deserves further studies. 

Other non-motor symptoms present in PD, such as constipation and other alterations in gastrointestinal motility mediated via catecholaminergic mechanisms [80,140], might also be alleviated by Atremorine. However, further investigation is needed on the central and peripheral effects of Atremorine, especially taking into account that the effects of Atremorine are genotype-related, involving both pathogenic genes associated with neurodegeneration, and genes of the cytochrome P450 family associated with drug metabolism [17,19].

All these data together clearly indicate that Atremorine is a very safe bioproduct in PD, with a powerful effect on catecholamines, especially dopamine and, to a lesser extent, noradrenaline. The effect of Atremorine on adrenaline is very modest, and no effect on serotonin levels can be detected one hour after oral administration. The effect of Atremorine on prolactin and GH is likely to result from the primary consequence of hypothalamic regulation mediated via dopaminergic and noradrenergic neurotransmission, and secondarily as a direct effect on the pituitary gland. In contrast, the effect on cortisol might result primarily from a direct effect on the adrenal gland, and secondarily from the hypothalamus–hypophyseal regulation of ACTH. According to these results, Atremorine may help to optimize neuroendocrine function in PD, especially in those patients with somatotropinergic, lactotropinergic and corticotropinergic dysregulation. The effects of Atremorine on plasma catecholamines might also be beneficial for PD patients with cardiovascular dysautonomia.

**Figure 3:**
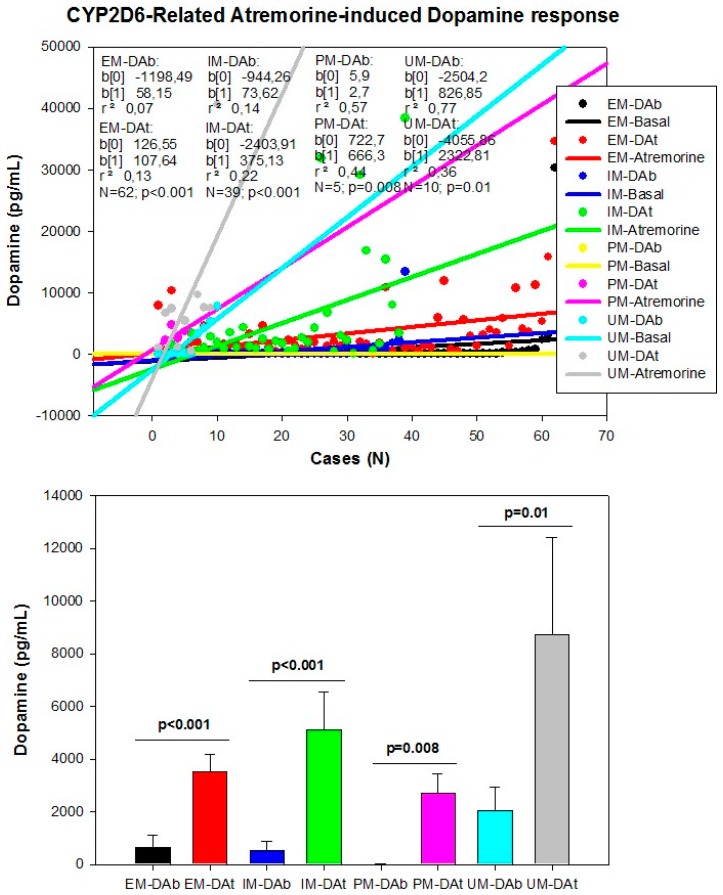
CYP2D6-related Atremorine-induced dopamine response. Basal (DAb) and Atremorine-induced dopamine response (DAt) in CYP2D6 extensive (EM), intermediate (IM), poor (PM), and ultra-rapid metabolizers (UM). Adapted with permission from Cacabelos et al. [17].

**Figure 4:**
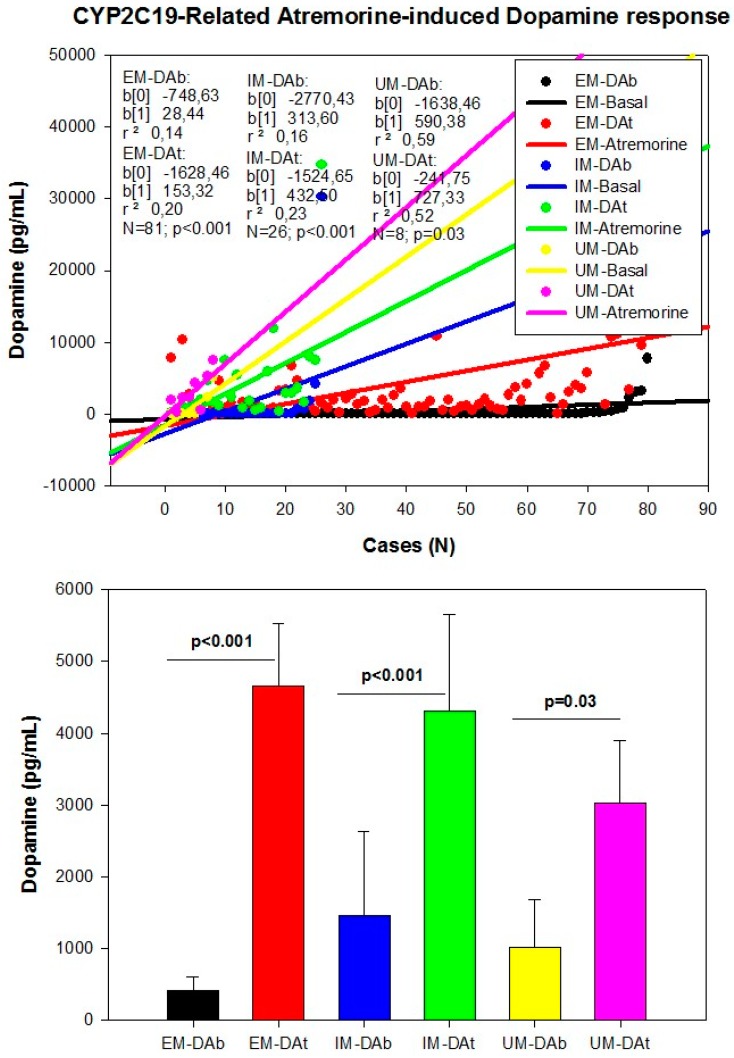
CYP2C19-related Atremorine-induced dopamine response. Basal (DAb) and Atremorine-induced dopamine response (DAt) in CYP2C19 extensive (EM), Intermediate (IM), and Ultra-rapid metabolizers (UM). Adapted with permission from Cacabelos et al. [17].

**Figure 5:**
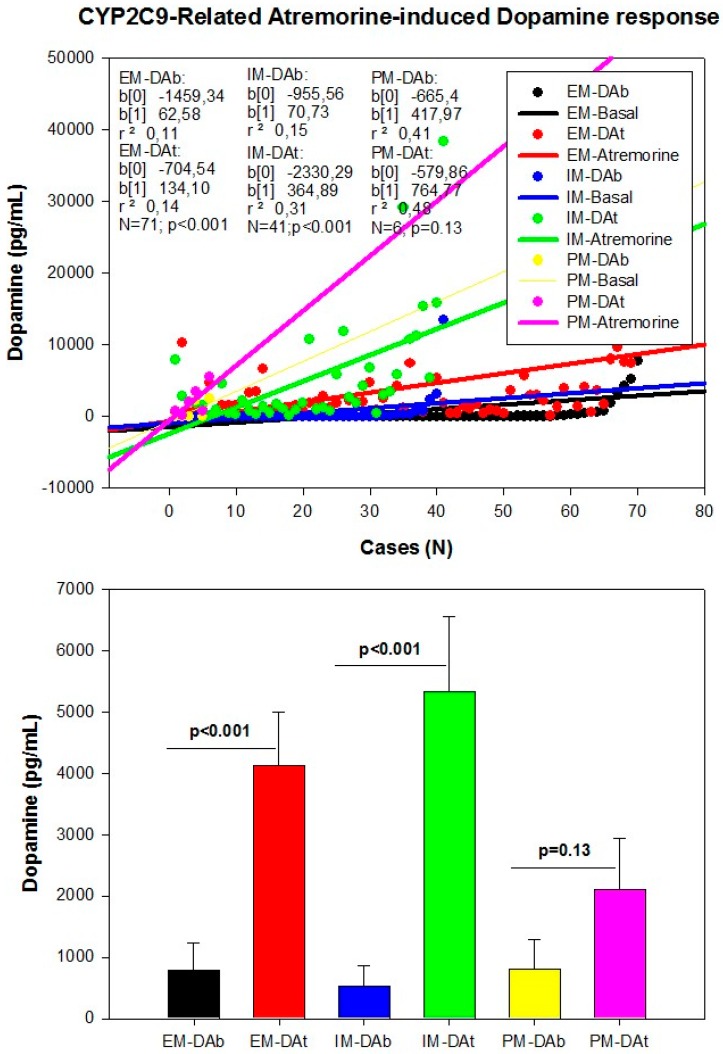
CYP2C9-related Atremorine-induced dopamine response. Basal (DAb) and Atremorine-induced dopamine response (DAt) in CYP2C9 extensive (EM), Intermediate (IM), and Poor metabolizers (PM). Adapted with permission from Cacabelos et al. [17].

**Figure 6:**
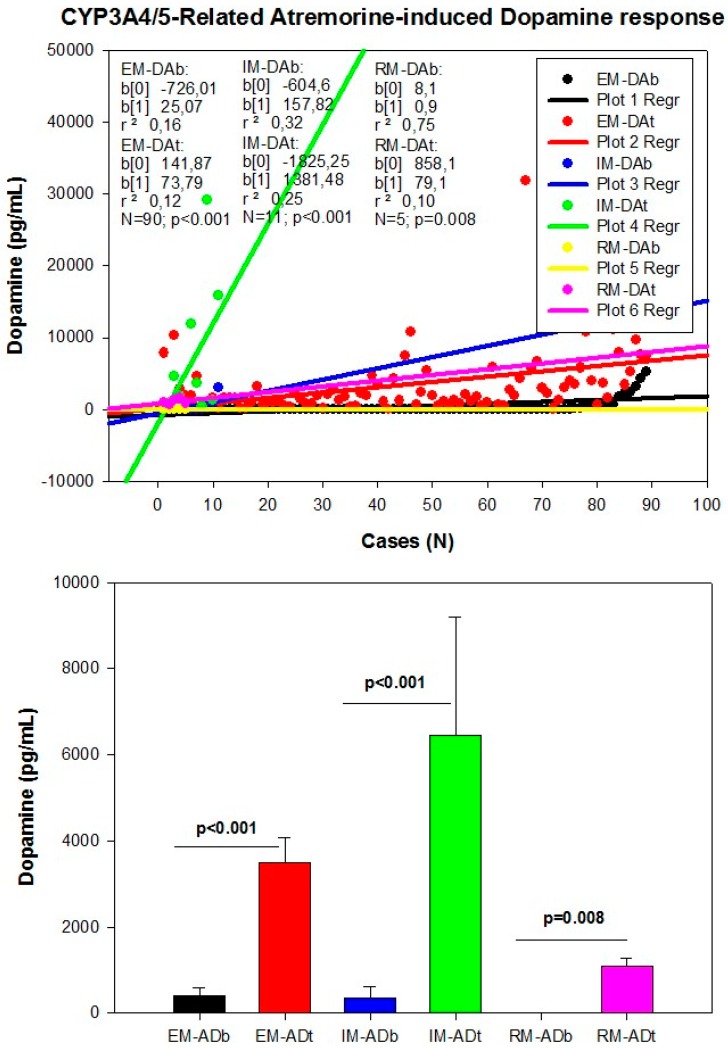
CYP3A4/5-related Atremorine-induced dopamine response. Basal (DAb) and Atremorine-induced dopamine response (DAt) in CYP3A4/5 extensive (EM), intermediate (IM), and rapid metabolizers (RM). Adapted with permission from Cacabelos et al. [17].

However, although the dopaminergic surge induced by Atremorine is proportional to basal DA levels in PD patients, with a potential 200–500-fold increase over basal levels, its real potency and pharmacodynamic and pharmacokinetic properties are highly influenced by genetic and pharmacogenetic factors [17] (Figure 3, Figure 4, Figure 5 and Figure 6). The condition of extensive (EM), intermediate (IM), poor (PM) or ultra-rapid metabolizer (UM) associated with different CYP variants, and the inheritance of the *APOE-4* allele as well, influence the Atremorine-induced dopamine response in PD patients [17]. Although practically 100% of the patients respond to Atremorine, the magnitude of the response is modulated by the pharmacogenetic profile of each patient. For instance, in absolute values, CYP2D6-PMs exhibit the lowest basal dopamine levels and a response to Atremorine which is lower than that of CYP2D6-EMs or IMs; however, CYP2D6-UMs show the highest basal dopamine levels and the most spectacular response to Atremorine [17] (Figure 3). The three major CYP2C19 genophenotypes show a similar response to Atremorine (Figure 4); in contrast, comparatively, CYP2C9-IMs (Figure 5) and CYP3A4/5-IMs (Figure 6) are the best responders and CYP2C9-PMs and CYP3A4/5-RMs are the worst responders to Atremorine, though all genophenotypes respond with a significant surge in dopamine levels one hour after Atremorine administration (5 g, p.o.) [17].

## 6. Further Considerations

Cardiovascular dysfunction is a common finding in PD patients. When PD patients are stratified by their cardiovascular function, as assessed with electrocardiogram (EKG), 39.50% show an abnormal EKG, 19.32% are borderline, and 41.18% exhibit a normal EKG [141].

Cardiac parasympathetic dysfunction occurs in the early phase of PD, but not necessarily in parallel with cardiac sympathetic dysfunction [88]. Low plasma levels of noradrenaline and adrenaline, secondary to the loss of catecholaminergic neurons in the rostral ventrolateral medulla, together with loss of nigral dopaminergic neurons, may be responsible for reduced sympathetic activity [142] and cardiovascular dysautonomia in PD [117]. Parkinson’s disease patients with abnormal EKG have lower levels of dopamine, and the basal levels of noradrenaline are substantially different between cases with abnormal vs normal EKG [18,141]. The administration of Atremorine tends to increase the plasma levels of the three catecholamines, with the highest impact on dopamine levels, and a minimum effect on adrenaline levels [18]. Basal biochemical conditions and cardiovascular function have been evaluated in PD patients without any treatment at the moment of their first diagnosis, and in PD patients chronically treated with antiparkinsonian drugs for more than one year. Both groups exhibited substantial biochemical and cardiovascular differences. In addition to changes in blood biochemical (i.e., urea, creatinine, phosphorous, alkaline phosphatase, folate, cyanocobalamin) and hematological parameters (neutrophils, basophils) [17], the most significant differences were found in plasma neurotransmitters and hormones. For instance, as expected, basal dopamine levels in untreated patients were below 20 pg/mL (11.22 ± 0.29 pg/mL; mean ± standard error (SE)) whereas basal dopamine levels in treated patients ranged from 21 to 30,000 pg/mL (2139.23 ± 804.72 pg/mL; mean ± SE) (*p* < 0.001) [17,18]. Chronic treatment with antiparkinsonian drugs reduces the levels of plasma serotonin, follicle-stimulating hormone (FSH), luteinizing hormone (LH) and estrogen, and tends to increase the levels of adrenaline, histamine, ACTH, cortisol, and testosterone in a non-significant mode [18]. Changes in neurotransmitters and endocrine parameters are highly influenced by the genomic profile of each patient [17,18,19].

Since PD patients undergo long-term periods of treatment with different antiparkinsonian drugs, which chronically alter neurotransmitter levels and hormonal regulation, some reflections are pertinent: (i) it is likely that the chronic overstimulation of the dopaminergic system at central and peripheral levels may become deleterious for cardio-cerebrovascular function and hormonal regulation; (ii) since the central dopaminergic dysfunction precedes the clinical onset of the disease by several decades, it is urgent to identify predictive biomarkers to protect the population at risk of suffering PD; (iii) the progression of PD over the past 50 years, in terms of gradual increase in prevalence and incidence rates, indicates that environmental toxicity may contribute to accelerating selective dopaminergic neurodegeneration; therefore, a better scrutiny of environmental risk factors is necessary to implement preventive programs in susceptible persons; (iv) most post-mortem neurochemical studies might be contaminated by chronic polypharmacy; (v) novel drugs and bioproducts for the treatment of PD should address dopaminergic neuroprotection to reduce premature neurodegeneration rather than dopaminergic overstimulation; and (vi) since biochemical changes and therapeutic outcomes are highly dependent upon the genomic profiles of PD patients, personalized treatments should rely on pharmacogenetic procedures to optimize therapeutics [16].

Modern neuroscience must embrace the idea that most brain disorders require more neuroprotection and fewer symptomatic repressors. Unfortunately, the history of neuropsychopharmacology is a history of chemical symptomatic repression with delayed consequences for patients and society in terms of chronic disability, family burden, and health costs. In the particular case of Parkinson’s disease, future challenges are (i) a better insight into the pathogenesis of premature dopaminergic neurodegeneration; (ii) the identification of biomarkers for an early diagnosis; (iii) the implementation of preventive programs to halt disease progression at pre-symptomatic stages; (iv) the development of novel antiparkinsonian drugs with specific neuroprotective effects on the dopaminergic system; and (v) the personalized treatment of PD patients.

## Figures and Tables

**Table 1:** Pharmacogenetics of antiparkinsonian drugs.

**Dopamine Precursors**		
**Drug**	**Properties**	**Pharmacogenetics**
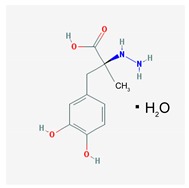	Name: Carbidopa; 28860-95-9; Lodosyn. IUPAC Name: Benzenepropanoic acid, α-hydrazino-3,4-dihydroxy-α-methyl-, monohydrate,(*S*) Molecular Formula: C_10_H_14_N_2_O_4_ H_2_O Molecular Weight: 244.24 g/mol Mechanism: Carbidopa is a peripheral decarboxylase inhibitor with little or no pharmacological activity when given alone in usual doses. It inhibits the peripheral decarboxylation of levodopa to dopamine. At the same time, reduced peripheral formation of dopamine reduces peripheral side effects, notably nausea or vomiting, and cardiac arrhythmias, although the dyskinesias and adverse mental effects associated with levodopa therapy tend to develop earlier. Effect: Antiparkinsonian agents. Dopamine precursors.	Pathogenic genes: *BDNF*, *PARK2* Mechanistic genes: *DRD2*, *OPRM1* Metabolic genes: Substrate: *COMT*, *DDC* Pleiotropic genes: *ACE*, *ACHE*
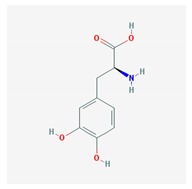	Name: Levodopa; 59-92-7; Levodopa; l-DOPA; Dopar; Bendopa; Dopasol; 3,4-dihydroxy-l-phenylalanine; Madopar. IUPAC Name: l-Tyrosine-3-hydroxy Molecular Formula: C_9_H_11_NO_4_ Molecular Weight: 197.19 g/mol Mechanism: Levodopa circulates in the plasma to the blood–brain–barrier, where it crosses, to be converted by striatal enzymes to dopamine. Carbidopa inhibits the peripheral plasma breakdown of levodopa by inhibiting its carboxylation, and thereby increases available levodopa at the blood–brain–barrier. Effect: Antiparkinsonian agents. Dopamine precursors.	Pathogenic genes: *ANKK1*, *BDNF*, *LRRK2*, *PARK2* Mechanistic genes: *CCK*, *CCKAR*, *CCKBR*, *DRD1*, *DRD2*, *DRD3*, *DRD4*, *DRD5*, *GRIN2A*, *GRIN2B*, *HCRT*, *HOMER1*, *LMO3*, *OPRM1* Metabolic genes: Substrate: *COMT*, *CYP1A2*, *CYP2B6*, *CYP2C19*, *CYP2D6*, *CYP3A4*, *CYP3A5*, *DBH*, *DDC*, *G6PD*, *MAOB*, *TH*, *UGT1A1*, *UGT1A9* Transporter genes: *SLC22A1*, *SLC6A3* Pleiotropic genes: *ACE*, *ACHE*
**Dopaminergic Agonists**		
**Drug**	**Properties**	**Pharmacogenetics**
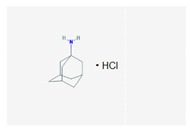	Name: Amantadine; 768-94-5; Amantadine; Symmetrel; PK-Merz; Amantadina. IUPAC Name: Tricyclo[3.3.1.1^3,7^]decan-1-amine, hydrochloride Molecular Formula: C_10_H_17_NHCl Molecular Weight: 187.71 g/mol Mechanism: Antiparkinsonian activity may be due to inhibition of dopamine reuptake into presynaptic neurons or by increasing dopamine release from presynaptic fibers. Effect: Antiparkinsonian agents; Adamantanes; Dopamine agonists.	Pathogenic genes: *PARK2* Mechanistic genes: *CCR5*, *CXCR4*, *DRD1*, *DRD2*, *GRIN3A* Metabolic genes: Substrate: *COMT*, *CYP1A2*, *CYP2B6*, *CYP2C19*, *CYP2D6*, *CYP3A4*, *CYP3A5*, *DDC*, *UGT1A1*, *UGT1A9* Transporter genes: *SLC22A1*
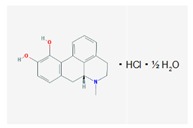	Name: Apomorphine; 58-00-4; Apomorhin; Apo-go; Apofin; Apokinon; Apokyn; Apomorfina. IUPAC Name: 4H-dibenzo[de,g]quinoline-10,11-diol, 5,6,6a,7-tetrahydro-6-methyl-hydrochloride,hemihydrate. Molecular Formula: C_17_H_17_NO_2_HCl1/2H_2_O Molecular Weight: 312.79 g/mol Mechanism: Stimulates postsynaptic D_2_-type receptors within the caudate putamen in the brain. Effect: Antiparkinsonian agents; Non-ergot-derivative dopamine receptor agonists.	Pathogenic genes: *PARK2* Mechanistic genes: *ADRA2A*, *ADRA2B*, *ADRA2C*, *CALY*, *DRD1*, *DRD2*, *DRD3*, *DRD4*, *DRD5*, *HTR1A*, *HTR1B*, *HTR1D*, *HTR2A*, *HTR2B*, *HTR2C* Metabolic genes: Substrate: *COMT*, *CYP1A2* (*minor*), *CYP2B6*, *CYP2C9* (*minor*), *CYP2C19* (*minor*), *CYP2D6*, *CYP3A4* (*minor*), *CYP3A5*, *DDC*, *UGT1A1*, *UGT1A9* Inhibitor: *CYP1A2* (*weak*), *CYP2C19* (*weak*), *CYP3A4* (*weak*)
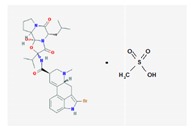	Name: Bromocriptine; 25614-03-3; Parlodel; Pravidel; Cycloset; Corpadel; Broman; Bromocriptina. IUPAC Name: Ergotaman-3′-6′-18-trione, 2-bromo-12′-hydroxy-2′-(1-methylethyl)-5′-(2-methylpropyl),monomethanesulfonate,(5′α). Molecular Formula: C_32_H_40_BrN_5_O_5_CH_4_SO_3_ Molecular Weight: 750.70 g/mol Mechanism: Semisynthetic ergot alkaloid derivative and dopamine receptor agonist which activates postsynaptic dopamine receptors in the tuberoinfundibular (inhibiting pituitary prolactin secrection) and nigrostriatal pathways (enhancing coordinated motor control). Causes transient increases in growth hormone secretion in individuals with normal growth hormone concentrations. Paradoxically causes sustained suppression of growth hormone secretion in acromegaly. Dysregulation of brain serotonine activity may also occur. Effect: Antiparkinsonian agents; Ergot-derivative dopamine receptor agonists.	Pathogenic genes: *ANKK1*, *BDNF*, *GSK3B*, *LRRK2* Mechanistic genes: *ABCB1*, *AKT1*, *BDNF*, *CCK*, *CCKAR*, *CCKBR*, *CNR1*, *DRD1*, *DRD2*, *DRD3*, *DRD4*, *DRD5*, *GRIN2A*, *GRIN2B*, *GSK3B*, *HCRT*, *HOMER1*, *LMO3*, *OPRM1* Metabolic genes: Substrate: *COMT*, *CYP1A2*, *CY22B6*, *CYP2C19*, *CYP2D6*, *CYP3A4* (*major*), *CYP3A5*, *DDC*, *MAOB*, *UGT1A1*, *UGT1A9* Inhibitor: *CYP1A2* (*weak*), *CYP3A4* (*moderate*) Transporter genes: *SLC22A1*, *SLC6A3* Pleiotropic genes: *ACE*, *APOE*
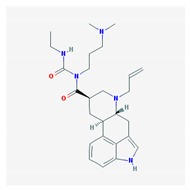	Name: Cabergoline; 81409-90-7; Cabergoline; Dostinex, Cabaser; Cabergolinum; Cabaseril; Cabergolina. IUPAC Name: Ergoline-8β-carboxamide, *N*-[3-(dimethylamino)propyl]-*N*-[(ethylamino)carbonil]-6-(2-propenyl) Molecular Formula: C_26_H_37_N_5_O_2_ Molecular Weight: 451.60 g/mol Mechanism: A long-acting dopamine receptor agonist. Has high binding affinity for dopamine D_2_-receptors and lesser affinity for D_1_, α_1_- and α_2_-adrenergic, and serotonin (5-HT_1_ and 5-HT_2_) receptors. Reduces serum prolactin concentrations by inhibiting release of prolactin from the anterior pituitary gland (agonist activity at D_2_ receptors). Effect: Antiparkinsonian agents; Ergot-derivative dopamine receptor agonists.	Pathogenic genes: *BDNF*, *GSK3B* Mechanistic genes: *ADRA2A*, *ADRA2B*, *ADRA2C*, *AKT1*, *BDNF*, *CNR1*, *DRD1*, *DRD2*, *DRD3*, *DRD4*, *DRD5*, *GSK3B*, *HTR1A*, *HTR1B*, *HTR1D*, *HTR2A*, *HTR2B*, *HTR2C*, *HTR7* Metabolic genes: Substrate: *COMT*, *CYP1A2*, *CYP2B6*, *CYP2C19*, *CYP2D6*, *CYP3A4* (*minor*), *CYP3A5*, *DDC*
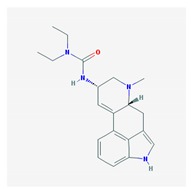	Name: Lisuride; 18016-80-3; Dopergin; Arolac; Dopergine; Dipergon; Lysenyl; Lisurida. IUPAC Name: 3-(9,10-didehydro-6-methylergolin-8α-yl)-1,1-diethylurea Molecular Formula: C_20_H_26_N_4_O Molecular Weight: 338.45 g/mol Mechanism: Displays dopaminergic, and consequently prolacting-reducing properties. Active substance lisuride has pronounced affinity for dopamine receptors in striatum and pituitary. Effect: Antiparkinsonian agents; Ergot-derivative dopamine receptor agonists. Antimigraine agents. Miscellaneous.	Mechanistic genes: *ADRA2A*, *ADRA2B*, *ADRA2C*, *DRD1*, *DRD2*, *DRD3*, *DRD4*, *DRD5*, *HTR1A*, *HTR1B*, *HTR1D*, *HTR2A*, *HTR2B*, *HTR2C* Metabolic genes: Substrate: *COMT*, *CYP1A2*, *CY22B6*, *CYP2C19*, *CYP2D6* (*major*), *CYP3A4* (*major*), *CYP3A5*, *DDC*, *UGT1A1*, *UGT1A9*
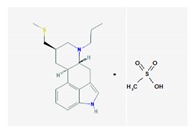	Name: Pergolide; 66104-22-1; Pergolide; Permax; Pergolida; Pergolidum. IUPAC Name: Ergoline,8-[(methylthio)methyl]-6-monomethenesulfonate Molecular Formula: C_19_H_26_N_2_SCH_4_O_3_S Molecular Weight: 410.59 g/mol Mechanism: A dopamin receptor agonist. Relieves symptoms of parkinsonism, presumably by directly stimulating postsynaptic dopamine receptors in corpus striatum. Reduces serum prolactine concentrations by inhibiting release of prolactin from anterior pituitary gland. Causes transient increase in serum somatotropin (growth hormone) concentrations and decreases in serum luteinizing hormone concentrations. Effect: Antiparkinsonian agents; Ergot-derivative dopamine receptor agonists.	Mechanistic genes: *ADRA1A*, *ADRA1B*, *ADRA1D*, *ADRA2A*, *ADRA2B*, *ADRA2C*, *DRD1*, *DRD2*, *DRD3*, *DRD4*, *DRD5*, *HTR1A*, *HTR1B*, *HTR1D*, *HTR2A*, *HTR2B*, *HTR2C* Metabolic genes: Substrate: *COMT*, *CYP1A2*, *CY22B6*, *CYP2C19*, *CYP2D6*, *CYP3A4* (*major*), *CYP3A5*, *DDC*, *UGT1A1*, *UGT1A9* Transporter genes: *SLC6A4*
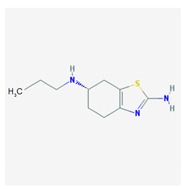	Name: Pramipexole; 104632-26-0; Pramipexole; Pramipexol; Parmital; Mirapex; Mirapexin; Sifrol IUPAC Name: 2,6-benzothiazolediamine, 4,5,6,7-tetrahydro-*N*^6^-propyl-,(*S*) Molecular Formula: C_10_H_17_N_3_S Molecular Weight: 211.33 g/mol Mechanism: By binding to D_2_ subfamily dopamine receptor, and to D_3_, and D_4_ receptors, it is though that Pramipexole can stimulate dopamine activity on nerves of striatum and substantia nigra. Effect: Antiparkinsonian agents; Non-ergot-derivative dopamine receptor agonists.	Pathogenic genes: *ANKK1*, *BDNF*, *LRRK2* Mechanistic genes: *ADRA2A*, *ADRA2B*, *ADRA2C*, *CCK*, *CCKAR*, *CCKBR*, *DRD1*, *DRD2*, *DRD3*, *DRD4*, *DRD5*, *GRIN2A*, *GRIN2B*, *HCRT*, *HOMER1*, *HTR1A*, *HTR1B*, *HTR1D*, *HTR2A*, *HTR2B*, *HTR2C*, *LMO3*, *OPRM1* Metabolic genes: Substrate: *COMT*, *CYP1A2*, *CY22B6*, *CYP2C19*, *CYP2D6*, *CYP3A4*, *CYP3A5*, *DDC*, *MAOB*, *UGT1A1*, *UGT1A9* Transporter genes: *SLC22A1*, *SLC6A3* Pleiotropic genes: *ACE*, *APOE*
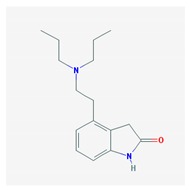	Name: Ropinirole; 91374-21-9; Ropinirole; ReQuip; Ropinirol; Ropinilorum; ReQuip CR IUPAC Name: 2-*H*-Indol-2-one 4-[2-(dipropylamino)ethyl]-1,3-dihydro-, monohydrochloride Molecular Formula: C_16_H_24_N_2_O Molecular Weight: 296.84 g/mol Mechanism: Has high relative in vitro specificity and full intrinsic activity at D_2_ and D_3_ dopamine receptor subtypes, binding with higher affinity to D_3_ than to D_2_ and D_4_ receptor subtypes. Although precise mechanism of action unknown, it is believed to be due to stimulation of postsynaptic dopamine D_2_-type receptors within caudate putamen in brain. Mechanism of Ropinirole-induced postural hypotension believed to be due to D_2_-mediated blunting of noradrenergic response to standing and subsequent decrease in peripheral vascular resistance. Effect: Antiparkinsonian agents; Non-ergot-derivative dopamine receptor agonists.	Pathogenic genes: *ANKK1*, *BDNF*, *LRRK2* Mechanistic genes: *ADRA2A*, *ADRA2B*, *ADRA2C*, *CCK*, *CCKAR*, *CCKBR*, *DRD1*, *DRD2*, *DRD3*, *DRD4*, *DRD5*, *GRIN2A*, *GRIN2B*, *HCRT*, *HOMER1*, *HTR1A*, *HTR1B*, *HTR1D*, *HTR2A*, *HTR2B*, *HTR2C*, *LMO3*, *OPRM1* Metabolic genes: Substrate: *COMT*, *CYP1A2* (*major*), *CY22B6*, *CYP2C19*, *CYP2D6*, *CYP3A4* (*minor*), *CYP3A5*, *DDC*, *MAOB*, *UGT1A1*, *UGT1A9* Inhibitor: *CYP1A2* (*moderate*), *CYP2D6* (*moderate*), *CYP3A4* (*moderate*) Transporter genes: *SLC22A1*, *SLC6A3* Pleiotropic genes: *ACE*, *APOE*
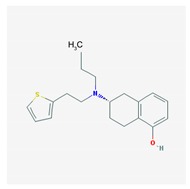	Name: Rotigotine; 99755-59-6; Rotigotine; Rotigotina; Neupro IUPAC Name: 1-Naphthalenol, 5,6,7,8-tetrahydro-6-[propyl[2-(2-thienyl)ethyl]amino]-6*S* Molecular Formula: C_19_H_25_NOs Molecular Weight: 315.47 g/mol Mechanism: A non-ergot dopamine receptor agonist with specificity for D_3_-, D_2_-, and D_1_-dopamine receptors. Although precise mechanism of action unknown of Rotigotine, it is believed to be due to stimulation of postsynaptic dopamine D_2_-type auto receptors within substantia nigra in brain, leading to improved dopaminergic transmission in motor areas in basal ganglia, notably caudate nucleus/putamen regions. Effect: Antiparkinsonian agents; Non-ergot-derivative dopamine receptor agonists.	Pathogenic genes: *ANKK1*, *BDNF*, *LRRK2* Mechanistic genes: *CCK*, *CCKAR*, *CCKBR*, *DRD1*, *DRD2*, *DRD3*, *DRD4*, *DRD5*, *GRIN2A*, *GRIN2B*, *HCRT*, *HOMER1*, *LMO3*, *OPRM1* Metabolic genes: Substrate: *COMT*, *MAOB* Transporter genes: *SLC22A1*, *SLC6A3* Pleiotropic genes: *ACE*, *APOE*
**Monoamine Oxidase B (MOB) Inhibitors**		
**Drug**	**Properties**	**Pharmacogenetics**
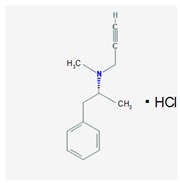	Name: Selegiline; 14611-51-9; Selegiline; Selegilina; l-Deprenalin; Emsam; Jumex; Eldepryl; Carbex IUPAC Name: Benzeneethanamine,*N*,α-dimethyl-*N*-2-propynyl-,hydrochloride,(*R*) Molecular Formula: C_31_H_17_NHCl Molecular Weight: 223.74 g/mol Mechanism: Potent, irreversible inhibitor of the monoamine oxidase (MAO). Plasma concentrations achieved via administration of oral dosage forms in recommended doses confer selective inhibition of the MAO type B, which plays a major role in metabolism of dopamine. Selegiline may also increase dopaminergic activity by interfering with dopamine reuptake at synapse. Effect: Antidepressants. Monoamine oxidase inhibitors. Antiparkinsonian agents. Monoamine oxidase B inhibitors.	Pathogenic genes: *ANKK1*, *BDNF*, *LRRK2* Mechanistic genes: *CCK*, *CCKAR*, *CCKBR*, *DRD1*, *DRD2*, *DRD3*, *DRD4*, *DRD5*, *GRIN2A*, *GRIN2B*, *HCRT*, *HOMER1*, *LMO3*, *OPRM1* Metabolic genes: Substrate: *COMT*, *CYP1A1*, *CYP1A2* (*minor*), *CYP1B1*, *CYP2A6* (*minor*), *CYP2B6* (*major*), *CYP2C8* (*minor*), *CYP2C19* (*major*), *CYP2D6* (*minor*), *CYP2E1* (*minor*), *CYP3A4* (*minor*), *CYP3A5*, *CYP19A1*, *DDC*, *MAOA*, *MAOB*, *UGT1A1*, *UGT1A9* Inhibitor: *CYP1A2* (*weak*), *CYP2A6* (*weak*), *CYP2C9* (*weak*), *CYP2C19* (*weak*), *CYP2D6* (*weak*), *CYP2E1* (*weak*), *CYP3A4* (*weak*), *MAOB* Transporter genes: *SLC22A1*, *SLC6A3* Pleiotropic genes: *ACE*, *APOE*
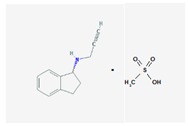	Name: Rasagiline; 136236-51-6; Azilet; Elbrux; Rasagilina; Raxac. IUPAC Name: 1*H*-Inden-1-amine, 2,3-dihydro-*N*-2-propynyl-,(*R*)-, methanesulfonate Molecular Formula: C_12_H_13_NCH_4_O_3_S Molecular Weight: 267.34 g/mol Mechanism: Potent, irreversible inhibitor of the MAO type B, which plays a major role in catabolism of dopamine. Inhibition of dopamine depletion in striatal region of brain reduces symptomatic motor deficits of Parkinson’s Disease. There is also experimental evidence of Rasagiline conferring neuroprotective effects (antioxidant, antiapoptotic), which may delay onset of symptoms and progression of neuronal deterioration. Effect: Antidepressants. Monoamine oxidase inhibitors. Antiparkinsonian Agents. Monoamine oxidase B inhibitors.	Pathogenic genes: *ANKK1*, *BDNF*, *LRRK2*, *PARK2* Mechanistic genes: *BLC2*, *CCK*, *CCKAR*, *CCKBR*, *DRD1*, *DRD2*, *DRD3*, *DRD4*, *DRD5*, *GRIN2A*, *GRIN2B*, *HCRT*, *HOMER1*, *LMO3*, *OPRM1* Metabolic genes: Substrate: *COMT*, *CYP1A2* (*major*), *CYP2B6*, *CYP2C19*, *CYP2D6*, *CYP3A4*, *CYP3A5*, *DDC*, *MAOB*, *UGT1A1*, *UGT1A9* Inhibitor: *MAOB* Transporter genes: *SLC22A1*, *SLC6A3* Pleiotropic genes: *ACE*, *APOE*
**Catecol-*O*-methyltransferase (COMT) Inhibitors**		
**Drug**	**Properties**	**Pharmacogenetics**
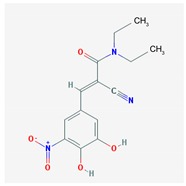	Name: Entacapone; 130929-57-6; Comtan; Comtess; Entacapona. IUPAC Name: *E*-α-cyano-*N*,*N*-diethyl-3,4-dihydroxy-5-nitrocinnamamida Molecular Formula: C_14_H_15_N_3_O_5_ Molecular Weight: 305.29 g/mol Mechanism: A selective and selective inhibitor of COMT. When entacapona is taken with levodopa, the pharmacokinetics are altered, resulting in more sustained levodopa serum levels compared to levodopa taken alone. Effect: Antiparkinsonian agents. Catechol-*O*-methyltransferase inhibitors.	Pathogenic genes: *ANKK1*, *BDNF*, *LRRK2*, *PARK2* Mechanistic genes: *CCK*, *CCKAR*, *CCKBR*, *DRD1*, *DRD2*, *DRD3*, *DRD4*, *DRD5*, *GRIN2A*, *GRIN2B*, *HCRT*, *HOMER1*, *LMO3*, *OPRM1* Metabolic genes: Substrate: *COMT*, *CYP1A2*, *CYP2B6*, *CYP2C19*, *CYP2D6*, *CYP3A4*, *CYP3A5*, *DDC*, *MAOB*, *UGT1A1*, *UGT1A3*, *UGT1A4*, *UGT1A6*, *UGT1A9*, *UGT2B7*, *UGT2B15* Inhibitor: *COMT*, *CYP1A2* (*weak*), *CYP2A6* (*weak*), *CYP2C9* (*weak*), *CYP2C19* (*weak*), *CYP2D6* (*weak*), *CYP2E1* (*weak*), *CYP3A4* (*weak*) Transporter genes: *SLC22A1*, *SLC6A3* Pleiotropic genes: *ACE*, *ACHE*, *APOE*
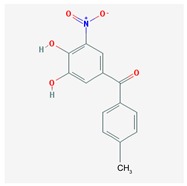	Name: Tolcapone; 134308-13-7; Tolcapona; Tasmar. IUPAC Name: Methanone, (3,4-hydroxy-5-nitrophenyl)(4-methylphenyl) Molecular Formula: C_14_H_11_NO_5_ Molecular Weight: 273.24 g/mol Mechanism: A selective and selective inhibitor of (COMT. In the presence of a decarboxylase inhibitor (e.g., carbidopa), COMT is the major degradation pathway for levodopa. Inhibition of COMT leads to more sustained plasma levels of levodopa and enhanced central dopaminergic activity. Effect: Antiparkinsonian agents. Catechol-*O*-methyltransferase inhibitors.	Pathogenic genes: *ANKK1*, *BDNF*, *LRRK2*, *PARK2* Mechanistic genes: *AKT1*, *CCK*, *CCKAR*, *CCKBR*, *CNR1*, *DRD1*, *DRD2*, *DRD3*, *DRD4*, *DRD5*, *GPT*, *GRIN2A*, *GRIN2B*, *GSK3B*, *HCRT*, *HOMER1*, *LMO3*, *OPRM1* Metabolic genes: Substrate: *COMT*, *CYP1A2*, *CYP2B6*, *CYP2C9*, *CYP2C19*, *CYP2D6*, *CYP3A4*, *CYP3A5*, *DDC*, *MAOB*, *UGT1A1*, *UGT1A3*, *UGT1A4*, *UGT1A6*, *UGT1A9*, *UGT2B7*, *UGT2B15* Transporter genes: *SLC22A1*, *SLC6A3* Pleiotropic genes: *ACE*, *APOE*

***ABCB1****:* ATP binding cassette subfamily B member 1; ***ACE****:* Angiotensin I converting enzyme; ***ACHE****:* Acetylcholinesterase; ***ADCY7****:* Adenylate cyclase 7; ***ADRA1A****:* Adrenoceptor α1A; ***ADRA1B****:* Adrenoceptor α1B; ***ADRA1D****:* Adrenoceptor α1D; ***ADRA2A****:* Adrenoceptor α2A; ***ADRA2B****:* Adrenoceptor α2B; ***ADRA2C****:* Adrenoceptor α2C; ***AKT1****:* v-Akt murine thymoma viral oncogene homolog 1; ***ANKK1****:* Ankyrin repeat and kinase domain containing 1; ***APOE****:* Apolipoprotein E; ***BDNF****:* Brain-derived neurotrophic factor; ***BLC2****:* B-cell chronic lymphocytic leukemia (CLL)/lymphoma 2; ***CALY****:* Calcyon neuron specific vesicular protein; ***CCK****:* Cholecystokinin, ***CCKAR****:* Cholecystokinin A receptor; ***CCKBR****:* Cholecystokinin B receptor; ***CCR5****:* C–C motif chemokine receptor 5 (gene/pseudogene); ***CHAT****:* Choline *O*-acetyltransferase; ***CNR1****:* Cannabinoid receptor 1 (brain); ***COMT****:* Catechol-*O*-methyltransferase; ***CREB1****:* cAMP responsive element binding protein 1; ***CXCR4****:* C–X–C motif chemokine receptor 4; ***CYP1A1****:* Cytochrome P450 family 1 subfamily A member 1; ***CYP1A2****:* Cytochrome P450 family 1 subfamily A member 2; ***CYP1B1****:* Cytochrome P450 family 1 subfamily B member 1; ***CYP2A6****:* Cytochrome P450 family 2 subfamily A member 6; ***CYP2B6****:* Cytochrome P450 family 2 subfamily B member 6; ***CYP2C19****:* Cytochrome P450 family 2 subfamily C member 19; ***CYP2C9****:* Cytochrome P450 family 2 subfamily C member 9; ***CYP2D6****:* Cytochrome P450 family 2 subfamily D member 6; ***CYP2E1****:* Cytochrome P450 family 2 subfamily E member 1; ***CYP3A4****:* Cytochrome P450 family 3 subfamily A member 4; ***CYP3A5****:* Cytochrome P450 family 3 subfamily A member 5; ***CYP19A1****:* Cytochrome P450 family 19 subfamily A member 1; ***DBH****:* Dopamine β-hydroxylase; ***DDC****:* DOPA decarboxylase; ***DRD1****:* Dopamine receptor D1; ***DRD2****:* Dopamine receptor D2; ***DRD3****:* Dopamine receptor D3; ***DRD4****:* Dopamine receptor D4; ***DRD5****:* Dopamine receptor D5; ***G6PD****:* Glucose-6-phosphate dehydrogenase; ***GPT****:* Glutamic-pyruvate transaminase (alanine aminotransferase); ***GRIN2A****:* Glutamate ionotropic receptor *N*-methyl-d-aspartate (NMDA) type subunit 2A; ***GRIN2B****:* Glutamate ionotropic receptor NMDA type subunit 2B; ***GRIN3A****:* Glutamate ionotropic receptor NMDA type subunit 3A; ***GSK3B****:* Glycogen synthase kinase 3 beta; ***HCRT****:* Hypocretin (orexin) neuropeptide precursor; ***HOMER1****:* Homer scaffolding protein 1; ***HRH1****:* Histamine receptor H1; ***HTR1A****:* 5-Hydroxytryptamine receptor 1A; ***HTR1B****:* 5-Hydroxytryptamine receptor 1B; ***HTR1D****:* 5-Hydroxytryptamine receptor 1D; ***HTR2A****:* 5-Hydroxytryptamine receptor 2A; ***HTR2B****:* 5-Hydroxytryptamine receptor 2B; ***HTR2C****:* 5-Hydroxytryptamine receptor 2C; ***HTR7****:* 5-Hydroxytryptamine receptor 7; ***LMO3****:* LIM domain only 3; ***LRRK2****:* Leucine-rich repeat kinase 2; ***MAOA****:* Monoamine oxidase A; ***MAOB****:* Monoamine oxidase B; ***OPRM1****:* Opioid receptor mu 1; ***PAH****:* phenylalanine hydroxylase; ***PARK2****:* Parkin RBR E3 ubiquitin protein ligase; ***SLC22A1****:* Solute carrier family 22 member 1; ***SLC6A3****:* Solute carrier family 6 member 3; ***SLC6A4****:* Solute carrier family 6 member 4; ***SST****:* Somatostatin; ***TH****:* Tyrosine hydroxylase; ***TSPO****:* Translocator protein; ***UGT1A1****:* UDP glucuronosyltransferase family 1 member A1; ***UGT1A3****:* UDP glucuronosyltransferase family 1 member A3; ***UGT1A4****:* UDP glucuronosyltransferase family 1 member A4; ***UGT1A6****:* UDP glucuronosyltransferase family 1 member A6; ***UGT1A9****:* UDP glucuronosyltransferase family 1 member A9; ***UGT2B7****:* UDP glucuronosyltransferase family 2 member B7; ***UGT2B15****:* UDP glucuronosyltransferase family 2 member B15. (Source: R. Cacabelos et al., [17,19]). IUPAC: International Union of Pure and Applied Chemistry.
